# Supplementary material for: Effectiveness and Safety of Ayurvedic Medicines in Type 2 Diabetes Mellitus Management: A Systematic Review and Meta-Analysis
Source: Front Pharmacol. 2022 Jun 8;13:821810. doi: 10.3389/fphar.2022.821810 (PMC9213670; doi:10.3389/fphar.2022.821810)
Supplement: Supplementary file 1 [file DataSheet1.zip › Supplementary Material/Forest and Funnel Plots/Syzygium cumini/HbA1c.pdf]

Syzygium cumini - HbA1c

Study	Intervention N	Intervention Mean	Intervention SD	Comparator N	Comparator Mean	Comparator SD	Mean Diff. with 95% CI	Weight (%)
Sahana 2010	15	8.00	1.43	10	7.30	1.00	0.70 [-0.32, 1.72]	45.08
Sidana 2016, Sidana 2017	50	8.31	1.40	49	9.03	1.31	-0.72 [-1.25, -0.19]	54.92
**Overall**							-0.08 [-1.46, 1.30]	

Heterogeneity: $\tau^2 = 0.83$, $I^2 = 82.79\%$, $H^2 = 5.81$

Test of $\theta_i = \theta_j$: Q(1) = 5.81, p = 0.02

Test of $\theta = 0$: z = -0.11, p = 0.91

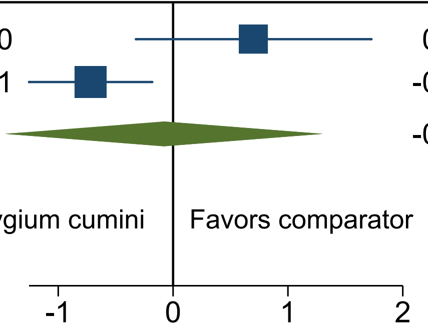

Random-effects REML model
